# Spectroscopic Studies on Unfolding Processes of Apo-Neuroglobin Induced by Guanidine Hydrochloride and Urea

**DOI:** 10.1155/2013/349542

**Published:** 2013-07-24

**Authors:** Cui Zhang, Chaohui Gao, Jianshuai Mu, Zhanglei Qiu, Lianzhi Li

**Affiliations:** School of Chemistry and Chemical Engineering, Liaocheng University, No. 1, Hunan Road, Liaocheng 252059, China

## Abstract

Neuroglobin (Ngb), a recently discovered globin, is predominantly expressed in the brain, retina, and other nerve tissues of vertebrates. The unfolding processes of apo-neuroglobin (apoNgb) induced by guanidine hydrochloride (GdnHCl) and urea were investigated by spectroscopic methods. In the unfolding processes, apoNgb's tertiary structural transition was monitored by the changes of intrinsic fluorescence emission spectra, and its secondary structural transition was measured by the changes of far-ultraviolet circular dichroism (CD) spectra. In addition, 8-anilino-1-naphthalenesulfonic acid (ANS), a hydrophobic cluster binding dye, was also used to monitor the unfolding process of apoNgb and to explore its intermediates. Results showed that GdnHCl-induced unfolding of apoNgb was via a three-state pathway, that is, Native state (*N*) → Intermediate state (*I*) → Unfolded state (*U*), during which the intermediate was inferred by an increase in fluorescence intensity and the change of CD value. Gibbs free energy changes are 10.2 kJ*·*mol^−1^ for the first unfolding transition and 14.0 kJ*·*mol^−1^ for the second transition. However, urea-induced unfolding of apoNgb only underwent a two-state transition: Native state (*N*) → Partially unfolded state (*P*). The result showed that GdnHCl can efficiently affect the conformational states of apoNgb compared with those of urea. The work will benefit to have an understanding of the unfolding mechanism of apoNgb induced by GdnHCl and urea.

## 1. Introduction

For a long time, hemoglobin (Hb) and myoglobin (Mb) have been considered as the only types of globin in vertebrates. Recently, a new globin, neuroglobin (Ngb), was discovered and added to the globin family in vertebrate [1]. Ngb is predominantly expressed in the brain, the retina, and other nerve tissues. Ngb is a protein composed of 151 amino acids with molecular weight of ~17000 Da [1, 2]. Although Ngb shares only 20–25% identity in amino acid sequence with vertebrate Hb and Mb, it displays the structural determinants of the globin fold [3]. Both Hb and Mb display a pentacoordinated heme, in which the iron ion is bound by four nitrogen atoms of the porphyrin ring and a proximal histidine in the F helix (His F8). Contrary to that, Ngb is a hexacoordinated globin in which the distal histidine E7 occupies the sixth coordination site in the absence of external ligands. Despite the fact that Ngb has been investigated for more than ten years, its exact physiological role is still uncertain [4]. In the past decades, Mb has been the subject of intensive structural and functional exploration under a variety of physiological and denaturing conditions [5–8]. The apo form of Mb (apoMb) has been investigated intensively and some studies showed that apoMb rendered different transition states under varying denaturing conditions [9–12]. Clearly, the deep study for apoNgb is also interesting, as it will lead to a better understanding pf its molecular features and biological functions. In our previous report, the pH-induced unfolding and refolding of apoNgb was studied by using spectroscopy [13]. Results revealed that apoNgb formed a folding intermediate known as molten globule, which possessed native-like secondary structure and lost its tertiary structure significantly at pH 2.0.

 To have a whole understanding of the process of protein folding, it is indispensible to investigate the structural and energetic information about transition-state intermediates. For this purpose, the conformation changes of transition-state intermediates existing in between the fully unfolded and the condensed native states have been studied. However, the intermediates of a protein unfolding emerge transiently and therefore are hard to study. One way to stabilize intermediates is to change the solvent conditions and then the intermediates could be the predominant forms. The frequently used solvent denaturants were guanidine hydrochloride (GdnHCl) and urea aqueous solutions. In this study, the unfolding process of apoNgb by GdnHCl and urea was studied through fluorescence and CD spectroscopy. Results showed that the unfolding process of apoNgb was a three-state pathway with an intermediate state in GdnHCl solution, whereas it underwent a simple two-state unfolding transition in urea solution.

## 2. Experiment

### 2.1. Materials

Yeast extract and tryptone were purchased from Oxoid Ltd. (England). Isopropyl-1-thio-dgalactopyranoside (IPTG) and dithiothreitol (DTT) were obtained from Sigma (USA). Ultrapure guanidine hydrochloride was purchased from Bio Basic Inc. (Canada). Urea and 8-Anilino-1-naphthalenesulfonic acid (ANS) were purchased from Sigma-Aldrich (USA). All other chemicals were of analytical grade and were used without further purification.

### 2.2. Methods

#### 2.2.1. Preparation of Neuroglobin and Apo-Neuroglobin

Ngb was expressed and purified as described in the literature [1, 14]. The purity of this protein was checked by 15% SDS-PAGE. Further, apoNgb was then prepared from Ngb by the acid-acetone method [13, 15]. The purity of the apoNgb was assessed spectrophotometrically; no significant absorption was observed in the Soret region of UV-Vis spectra. The concentration of apoNgb was determined by UV absorbance in 6 M guanidine hydrochloride using the method of Edelhoch [16].

#### 2.2.2. Spectroscopic Measurements

All stock solutions were prepared in 0.1 M phosphate buffer (pH = 7.0). GdnHCl and urea stock solution were prepared by weight and their concentrations were checked by refractive index measurement [17]. Denaturation experiments of apoNgb induced by GdnHCl and urea were performed as follows: desired concentration of protein and denaturant was obtained by mixing calculated amounts of the stock protein, buffer, and denaturant solutions. Mixed solutions were incubated at 25°C for 12 h to precede the reaction.

Both apoNgb intrinsic and ANS fluorescence emission spectra were recorded on an LS55 Luminescence spectrometer (PerkinElmer, USA). Excitation and emission slit widths were 10 nm, and the scan speed was 300 nm·min^−1^. The intrinsic fluorescence emission spectra were recorded from 310 to 420 nm using an excitation wavelength of 295 nm. The incubation time was 12 h when the fluorescence emission spectrum was recorded. For determining the binding of ANS to apoNgb at serial GdnHCl concentrations, ANS was added to a final concentration of 20 *μ*M to apoNgb samples that had been preincubated for 12 h in serial concentrations of GdnHCl, and then ANS fluorescence spectra were recorded after 30 min. The ANS fluorescence spectra were measured with an excitation wavelength of 350 nm, and the emission spectra were recorded from 400 to 600 nm. The ANS concentration was determined spectrophotometrically according to the molar absorption coefficient of 4950 M^−1^·cm^−1^ [18].

CD spectra of apoNgb were measured using a JASCO J-810 spectropolarimeter from 190 to 250 nm using a cylindrical quartz cell of path length 1 cm, with a 0.1 nm resolution, a response time of 1 s, and a scan speed of 100 nm/min. The optical system was protected by high pure N_2_ at 5 L/min flow rate. The spectra were averages of three consecutive scans, corrected by subtracting corresponding blanks, and subjected to noise reduction. 

## 3. Results and Discussion

The GdnHCl-induced and Urea-induced structural changes of apoNgb have been investigated by using fluorescence, ANS fluorescence, and far-UV CD spectroscopic techniques. Time-dependent changes of the structural parameters were monitored until an unfolding equilibrium was reached under these conditions. Results showed that an incubation of 12 h was sufficient to achieve the unfolding equilibrium of apoNgb under the conditions of denaturants.

### 3.1. GdnHCl-Induced Unfolding

#### 3.1.1. Fluorescence Spectra

**Figure 1:**
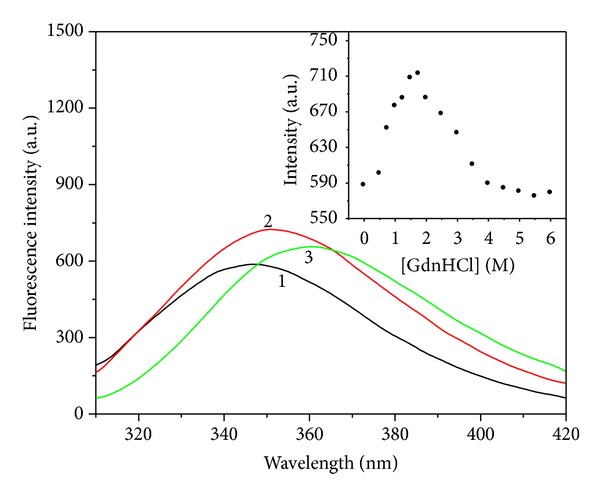
Fluorescence spectra of apoNgb at various GdnHCl concentrations. ApoNgb concentration was 2 *μ*M. From 1 to 3, the concentrations of GdnHCl were 0, 1.75, and 6 M, respectively. Inset: plot of emission intensity at 347 nm versus GdnHCl concentration.

**Figure 2:**
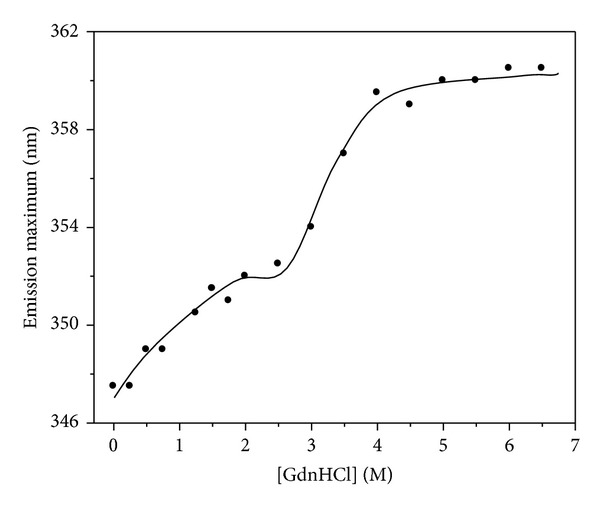
GdnHCl-induced unfolding profile of apoNgb monitored by maximal emission wavelength.

It is well known that the intrinsic fluorescence of proteins originated from the tryptophan (Trp) and tyrosine (Tyr) fluorophores is very sensitive to the microenvironments of the amino acid residues. Changes in emission spectra result from the conformational transitions, subunit association, substrate binding, or denaturation [19, 20]. The Ngb fluorescence is predominantly contributed by tryptophan (Trp) and tyrosine (Tyr) of this molecule. The Ngb protein has 3 Trp and 4 Tyr, and the maximal emission wavelength is at 338 nm, which indicates that there is only a small exposure to the protein surface among these Trp residues [14]. GdnHCl-induced unfolding of apoNgb was studied by fluorescence spectra; the tryptophan fluorescence was used here to probe structural changes of protein under unfolding. The apoNgb unfolding transition was monitored at different GdnHCl concentrations through the changes of fluorescence intensity and the maximal fluorescence wavelength.  Figure 1 showed the fluorescence spectra and fluorescence intensity of apoNgb at different GdnHCl concentration, and Figure 2 showed the changes of the *λ*_max⁡_ of apoNgb in the presence of increasing GdnHCl concentrations. The unfolding induced by GdnHCl clearly exhibited two transition processes. During the first transition, there was an increase in fluorescence intensity and a red-shift in the *λ*_max⁡_ as the concentration of GdnHCl increased from 0 to 1.75 M. These changes indicated the formation of an intermediate which had a higher fluorescence quantum yield than the native apoNgb. The fluorescence spectrum of this intermediate was slightly red-shifted, suggesting that the Trp residues environments were not appreciably altered. During the second transition, further denaturation process was accompanied by a decrease in the fluorescence intensity and a red-shift in the *λ*_max⁡_. This unfolding profile of apoNgb was similar to the unfolding of apoMb [10, 21]. The spectra showed a clear decrease in fluorescence intensity and a further red-shift from 351 nm to 361 nm was observed as the GdnHCl concentration was raised from 1.75 to 4 M. However, no further apparent changes were observed both in fluorescence emission maximum peak and intensity with further increasing of GdnHCl concentration. This fluorescence red-shift indicated that the Trp residues got more exposed to polar environment because the apoNgb's polypeptide chain was unfolded in the presence of GdnHCl.

#### 3.1.2. Circular Dichroism Spectra

**Figure 3:**
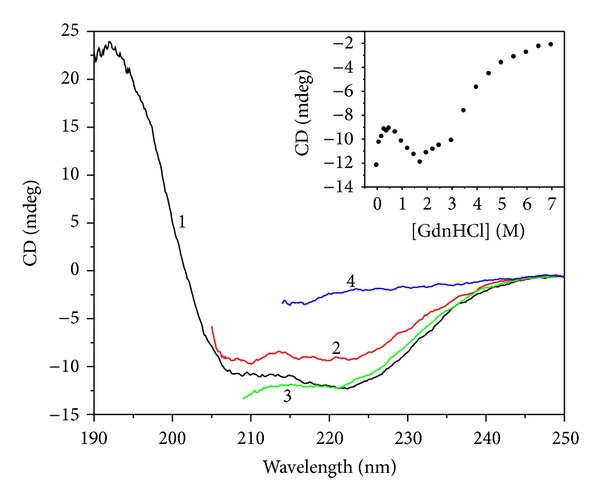
CD spectra of apoNgb at various GdnHCl concentrations. ApoNgb concentration was 7 *μ*M. 1–4: 0, 0.5, 1.75, 7 M GdnHCl. Inset showed GdnHCl-induced unfolding profile of apoNgb at 222 nm.

Proteins with different secondary structure have different absorption intensity and absorption position in the far-UV region of CD. Structural changes in proteins can be detected by monitoring their CD spectra, especially in the far-UV region where the main contribution to the spectra comes from the secondary structure of the peptide backbone. The far-UV CD spectra of apoNgb in the presence of serial GdnHCl concentrations were shown in Figure 3. It was shown that apoNgb exhibited a strong positive maximum at 192 nm and two negative minima at 208 nm and 222 nm, characteristic of the high *α*-helix content of the protein [14]. Incubation of apoNgb in GdnHCl solution resulted in the changes of the spectral shape and the loss of ellipticity of apoNgb. A three-step change of CD signal at 222 nm was observed with increasing concentrations of GdnHCl. The first transition occurred from 0 and 0.5 M GdnHCl and was followed by an increase of CD signal between 0.5 to 1.75 M, whereas the third transition occurred over 1.75 M GdnHCl. Approximately 25% of the CD signal at 222 nm was lost during the first transition, indicating partial unfolding of apoNgb molecules under these concentrations. However, the CD signal at 222 nm increased again during the second transition with further increase of GdnHCl concentration, getting similar value to that of the native state. Then, a complete loss of secondary structure happened during the third transition, showing that apoNgb adopted a complete unfolded state. This result is consistent with that of the previous fluorescence study; that is, apoNgb underwent an intermediate state in the unfolding process.

#### 3.1.3. ANS Binding

**Figure 4:**
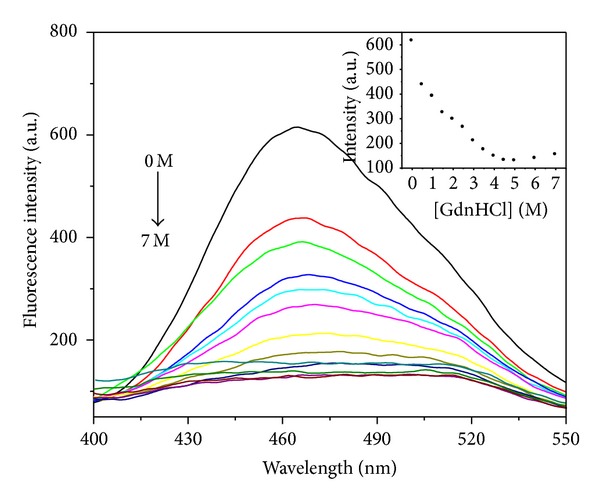
ANS fluorescence emission spectra in the presence of apoNgb at various GdnHCl concentrations. Inset: changes of fluorescence intensity at 465 nm. Concentrations of apoNgb and ANS were 2 *μ*M and 20 *μ*M, respectively.

The hydrophobic fluorescent dye ANS was widely used to probe the exposure of the hydrophobic region upon protein unfolding. The binding of ANS to hydrophobic regions of proteins resulted in a big enhancement of ANS fluorescence intensity and a significant blue-shift of the *λ*_max⁡_. We have studied the formation of molten globule-like intermediates during the unfolding process of apoNgb induced by pH change [13]. In order to investigate the intermediate states of apoNgb induced by GdnHCl, the binding of ANS to apoNgb in GdnHCl solution was performed.  Figure 4 showed the fluorescence emission spectra of ANS incubated with apoNgb at varying GdnHCl concentrations. The *λ*_max⁡_ of ANS incubated with native apoNgb was 465 nm, whereas the *λ*_max⁡_ of ANS exposed to free water was at 515 nm. Addition of ANS to apoNgb resulted in a blue-shift and enhancement in fluorescence intensity accompanied by an increase in quantum yield, indicating the binding of ANS molecules to the exposed hydrophobic patches in the native conformation of apoNgb. It appears quite likely that the ANS occupies the heme-binding site; the similar result was reported in the binding of ANS to apoMb [22]. During the unfolding process of apoNgb, the ANS fluorescence intensity decreased until 4 M GdnHCl. From 0 to 1.5 M GdnHCl, the ANS fluorescence intensity decreased by nearly 60%; the wavelength of maximum emission remained centered around 465 nm. When the denaturant concentrations were between 1.5 and 4 M, both of the fluorescence intensity and the wavelength of emission maximum changed. When GdnHCl is above 4 M, there were no obvious changes in the fluorescence intensity and the wavelength of maximum emission of ANS. The ANS fluorescence spectra exhibited a broad peak, similar to the ANS fluorescence spectrum in water, which indicated the transfer of the ANS molecules from a hydrophobic to a hydrophilic environment. This also suggested that apoNgb became unfolded when the ANS binding sites of apoNgb exposed to polar solvent. The ANS binding studies showed that the unfolding intermediate cannot induce other hydrophobic clusters which can be accessible to ANS. GdnHCl-induced unfolding of apoNgb did not induce an ANS-bound intermediate. The intermediate of apoMb observed in 1 M GdnHCl was a molten-globule intermediate [7]. However, unlike the intermediate of apoMb, the intermediate of apoNgb induced by 1.75 M GdnHCl does not exhibit a molten-globule type intermediate.

#### 3.1.4. Estimation of Gibbs Free Energy [23]

The GdnHCl-induced unfolding of apoNgb can be described as the following scheme:

(1) Native  state  (N)→  Intermediate  state  (I)→  Unfolded  state  (U).

Each equilibrium in such a scheme was classically fitted to a two-state process. The free energy of folding of protein at a given concentration of GdnHCl, Δ*G*_*d*_, can be defined as follows:

(2) $$\begin{array}{c} \Delta G_{d}=-RT\ln\left(\frac{F}{U}\right), \end{array}$$

where *F* represents the concentration of protein in the highly folded state and *U* represents the concentration of protein in the highly denatured state.

 The concentration of *F* and *U* can be calculated from the measurements of intrinsic fluorescence intensity or circular dichroism:

(3) $$\begin{array}{c} \frac{F}{U}=\frac{S_{F}-S}{S-S_{U}}, \end{array}$$

where *S* denotes the observed signal at various concentration of denaturant, *S*_*F*_ denotes the signal of the folded state, and *S*_*U*_ denotes the signal of the unfolded state. Free energy change for the first unfolding transition is 10.2 kJ·mol^−1^ and free energy change for the second transition is 14.0 kJ·mol^−1^. This indicated that it was easier to get equilibrium in the first unfolding transition than that in the second transition.

### 3.2. Urea-Induced Unfolding

#### 3.2.1. Fluorescence Spectra

**Figure 5:**
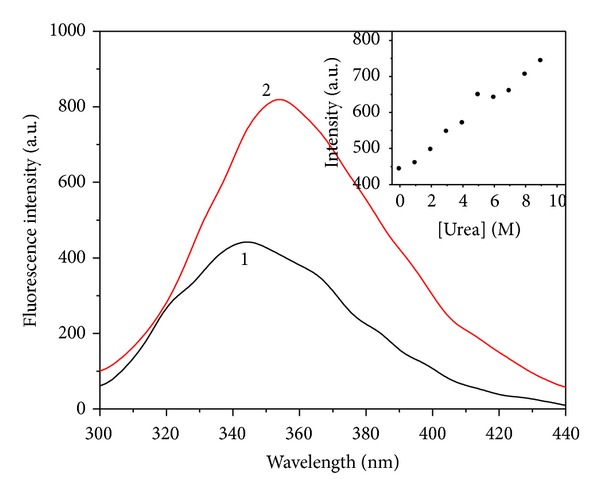
Fluorescence spectra of apoNgb at various urea concentrations. ApoNgb concentration was 2 *μ*M. The concentrations of urea were 0 M (1) and 9 M (2). Inset: plot of emission intensity at 346 nm versus urea concentration.

**Figure 6:**
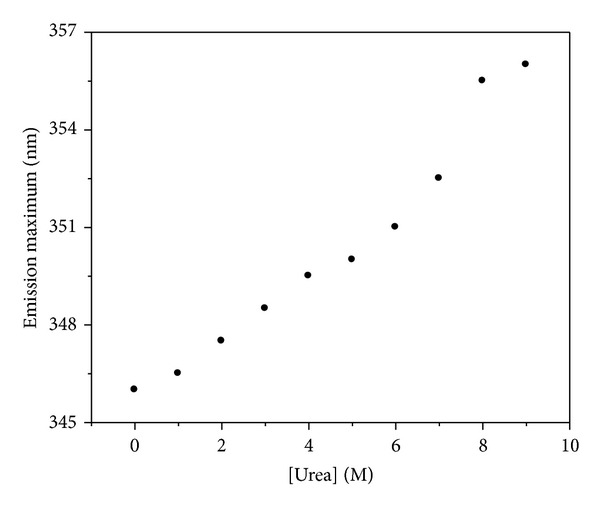
Urea-induced unfolding profile of apoNgb monitored by maximal emission wavelength.

Figure 5 showed the unfolding process of apoNgb in urea solution monitored by fluorescence spectra.  Figure 6 showed the *λ*_max⁡_ changes of apoNgb in the presence of urea with increasing concentrations. The urea-induced unfolding process was accompanied by an increase in fluorescence intensity and a red-shift to 356 nm, indicating gradual exposure process of tryptophan residues to aqueous surrounding. A red-shift to 361 nm was observed when GdnHCl was used for unfolding, showing that its tryptophan residues were almost fully exposed. The red-shifted maximum wavelength was 356 nm in the presence of urea, indicating that the tryptophan residues were not fully exposed to the solvent despite urea concentration was raised up to 9 M. GdnHCl and urea did not unfold apoNgb to the same extent. Results demonstrated that urea-induced unfolding of apoNgb was different from that of GdnHCl. In the presence of urea, apoNgb was unfolded only via a simple transition but not via any intermediate state.

#### 3.2.2. Circular Dichroism Spectra

**Figure 7:**
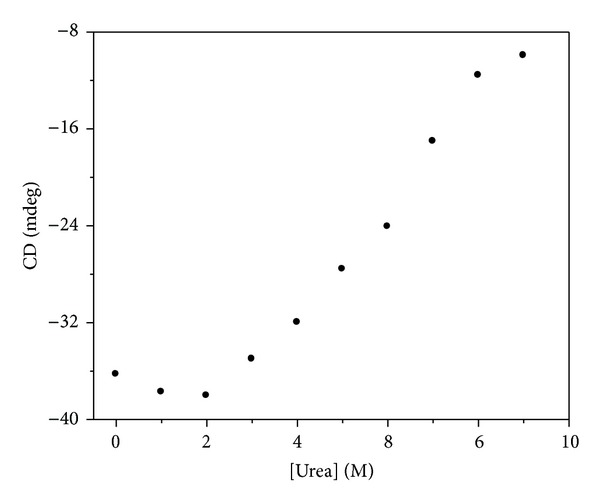
Urea-induced unfolding profile of apoNgb monitored by far-UV CD at 222 nm. ApoNgb concentration was 2 *μ*M.

The plotting of apoNgb ellipticities at 222 nm versus urea concentrations was shown in Figure 7. No obvious change in ellipticity was observed when urea is below 2 M; the molecular transition reflecting a disorganization of secondary structure occurred when urea is above 2 M. However, apoNgb can still maintain partially residual ellipticity at 9 M urea, whereas the GdnHCl-unfolded form cannot. The difference between urea-unfolded state and GdnHCl-unfolded state suggested that the existence of some residual secondary structure in the urea-unfolded states. This phenomenon indicated that apoNgb rendered a partially unfolded state in urea. Therefore, urea-induced unfolding can be represented by the following scheme:

(4) $$\begin{array}{c} \text{Native}\;\text{state}\;\left(N\right)\to \text{Partially}\;\text{unfolded}\;\text{state}\;\left(P\right). \end{array}$$

#### 3.2.3. ANS Binding

**Figure 8:**
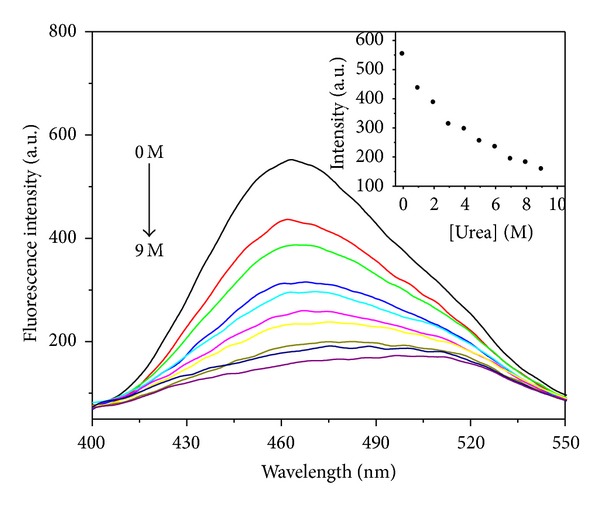
ANS fluorescence emission spectra in the presence of apoNgb at various urea concentrations. Inset: changes of fluorescence intensity at 465 nm. Concentrations of apoNgb and ANS were 2 *μ*M and 20 *μ*M, respectively.

The hydrophobic dye ANS was used to probe the exposure of hydrophobic region upon unfolding of apoNgb in urea solution (Figure 8). A sharp decrease in ANS fluorescence intensity was observed during the transition of apoNgb induced by increasing urea concentration; a red shift of ANS emission spectra can also be seen. The red shift reflected a higher degree of accessibility for water molecules to ANS binding region within apoNgb protein. When incubated with 9 M urea, ANS fluorescence spectrum was similar to that in water, indicating the transfer of the ANS molecules from a hydrophobic to a hydrophilic environment. However, ANS binding site (hydrophobic) was fully exposed to polar environment at 4 M GdnHCl. These results show that no other exposed hydrophobic clusters on structural sites were accessible to ANS. Therefore, the urea-induced unfolding of apoNgb does not lead to an ANS-bound intermediate.

## 4. Conclusions

Conformational changes of apoNgb in GdnHCl and urea have been studied in this work by spectroscopic techniques. Results showed that GdnHCl-induced unfolding of apoNgb is a three-state pathway through an intermediate state, Native state (*N*) → Intermediate state (*I*)→ Unfolded state (*U*). In a previous study, we have investigated the acid-induced structural transition of apoNgb. At pH 2.0, apoNgb has a compact globular state. In this work, it was found that the GdnHCl-induced unfolding of apoNgb is involved in the occurrence of an intermediate, which was observed when GdnHCl concentration is 1.75 M. The existence of such an intermediate was confirmed by a reduced hydrophobicity of the tryptophan microenvironments which was evidenced by fluorescence spectra, as well as by the fact of a high content of native-like secondary structure as showing in CD spectra. These two intermediates have similar secondary structure but differ in the tryptophanyl fluorescence emission and ANS binding. Therefore, the intermediate state induced by GdnHCl did not correspond to the compact globular state induced by acid.

In 9 M urea solution, the wavelength of fluorescence emission maximum underwent a red shift from 344 nm to 356 nm, but some residual secondary structure was still retained, indicating that apoNgb is a partially unfolded state in urea. Hence, urea-induced unfolding of apoNgb is a single transition and does not induce an intermediate state. The urea-induced denaturation pathway of apoNgb can be described as: Native state (*N*) → Partially unfolded state (*P*). In short, apoNgb underwent a two-state unfolding transition in urea solution. The different unfolding behavior of apoNgb in the two denaturants is because that GdnHCl is a stronger denaturant than urea, as well as GndHCl is charged while urea is neutral, which has a different effect on the stability of a protein.
